# Genome-Wide Investigation of the *PtrCHLP* Family Reveals That *PtrCHLP3* Actively Mediates Poplar Growth and Development by Regulating Photosynthesis

**DOI:** 10.3389/fpls.2022.870970

**Published:** 2022-05-10

**Authors:** Fang He, Yu-Jie Shi, Qi Chen, Jun-Lin Li, Meng-Xue Niu, Cong-Hua Feng, Meng-Meng Lu, Fei-Fei Tian, Fan Zhang, Tian-Tian Lin, Liang-Hua Chen, Qin-lin Liu, Xue-Qin Wan

**Affiliations:** ^1^Sichuan Province Key Laboratory of Ecological Forestry Engineering on the Upper Reaches of the Yangtze River, College of Forestry, Sichuan Agricultural University, Chengdu, China; ^2^College of Landscape Architecture, Sichuan Agricultural University, Chengdu, China; ^3^Beijing Advanced Innovation Center for Tree Breeding by Molecular Design, National Engineering Laboratory for Tree Breeding, College of Biological Sciences and Technology, Beijing Forestry University, Beijing, China

**Keywords:** poplar, *CHLP*, chlorophyll biosynthesis, photosynthesis, plant growth

## Abstract

Chlorophyll (Chl) plays a crucial role in plant photosynthesis. The geranylgeraniol reductase gene (*CHLP*) participates in the terminal hydrogenation of chlorophyll biosynthesis. Although there are many studies related to the genome-wide analysis of *Populus trichocarpa*, little research has been conducted on *CHLP* family genes, especially those concerning growth and photosynthesis. In this study, three *CHLP* genes were identified in *Populus*. The evolutionary tree indicated that the *CHLP* family genes were divided into six groups. Moreover, one pair of genes was derived from segmental duplications in *Populus*. Many elements related to growth were detected by *cis*-acting element analysis of the promoters of diverse *PtrCHLPs*. Furthermore, *PtrCHLPs* exhibit different tissue expression patterns. In addition, *PtrCHLP3* is preferentially expressed in the leaves and plays an important role in regulating chlorophyll biosynthesis. Silencing of *PtrCHLP3* in poplar resulted in a decrease in chlorophyll synthesis in plants, thus blocking electron transport during photosynthesis. Furthermore, inhibition of *PtrCHLP3* expression in poplar can inhibit plant growth through the downregulation of photosynthesis. Ultimately, *PtrCHLP3* formed a co-expression network with photosynthesis and chlorophyll biosynthesis-related genes, which synergistically affected the growth and photosynthesis of poplars. Thus, this study provides genetic resources for the improved breeding of fast-growing tree traits.

## Introduction

Chlorophyll plays a crucial role in photosynthetic organisms such as plants (Flood et al., 2011). Chlorophyll molecules are ubiquitous in these photosynthetic organisms and perform the process of capturing light energy in the antenna system by driving electron transport in the reaction center (Nagata et al., 2005). Chlorophyll a is the major electron donor in the reactive centers of PSI and PSII (Lin et al., 2014). With the development of molecular biology and protein structure, key members of the chlorophyll biosynthesis pathway have been fully explored and identified.

Chlorophyll biosynthesis is a complex process that begins with l-glutamyl-tRNA (Beale, 2005). Several enzymes and 27 genes are involved in this process (Beale, 2005; Nagata et al., 2005). Chlorophyll biosynthesis is a dynamic equilibrium process regulated by several genes, which can affect chlorophyll accumulation, photosynthetic capacity, chloroplast development, and leaf color (Mei et al., 2017; Ohmiya et al., 2019; Zhang et al., 2021). Many yellow-green leaf mutants are also closely related to genes involved in chlorophyll biosynthesis. In *lGL1* mutants, the expression levels of some key genes involved in chlorophyll biosynthesis and photosynthesis are significantly changed in rice, such as *ChlD*, *ChlI*, *Hema1*, *Ygl1*, *POR*, *Cab1R*, *Cab2R*, *PsaA*, and *rbcL* (Mei et al., 2017). In rice, the *YL-1* gene encodes magnesium protoporphyrin IX monomethyl ester cyclase, which is necessary for chlorophyll biosynthesis and chloroplast membrane stability (Sheng et al., 2017). In addition, it inhibits the expression of *HrHEMA*, *HrPOR*, and *HrCAO* genes in *Chrysanthemum*, resulting in leaf yellowing and chloroplast structural changes (Zhang et al., 2021).

The final stage of chlorophyll a (Chl a) biosynthesis is the esterification of the tetrapyrrole moiety and chlorophyllide (Havaux et al., 2003). Geranylgeranyl reductase (CHLP, EC: 1.3.1.111), a key enzyme in the final step of Chl a biosynthesis, participates in the terminal hydrogenation step of chlorophyll biosynthesis (Addlesee and Hunter, 2002; Zhang et al., 2015). In the last step of Chl a biosynthesis, chlorophyll synthase (CHL) catalyzes the esterification of chlorophyll (Chlide) with geranylgeranyl diphosphate (GGPP) or phytopyrophosphate (PPP) to form geranylgeranylated Chl a (Chl aGG) or phytylated Chl a, respectively (Shpilyov et al., 2005). In turn, CHLP catalyzes the reduction of free GGPP to PPP, and Chl aGG is reduced to phytyl-chlorophyll a, thus producing chlorophyll a (Addlesee and Hunter, 2002; Shpilyov et al., 2005).

*CHLP* genes have been identified in many species, including photosynthetic bacteria, algae, tobacco, rice, peaches, olives, and tomatoes (Addlesee and Hunter, 2002; Shpilyov et al., 2005; Deng et al., 2014; Zhang et al., 2015; Tan et al., 2019). Addlesee et al. (1996) discovered that the *CHLP* of the cyanobacterium *Synechocystis* sp. PCC 6803 can complement the function of the *bchp* mutant of *R. sphaeroides*. In 2005, Alexey studied a *chlp* mutant of the cyanobacterium *Synechocystis* sp. PCC 6803 and found that the cell chlorophyll and carotenoid content were decreased in the mutant, and the photosystem became unstable and could not grow autotrophically (Shpilyov et al., 2005). Plant growth was slow after *CHLP* gene silencing in tobacco, and the leaves showed a pale or mottled phenotype (Tanaka et al., 1999). Some *chlp* mutants have also been identified in *Oryza sativa*. The *lyl1-1* mutant has a dynamic yellow-green leaf, with reduced chlorophyll content, inhibited chloroplast development, and sensitivity to light (Zhou et al., 2013). Similarly, the growth rate of the yellow-green leaf *502ys* mutant was slowed, and the development of chlorophyll and chloroplasts was consistent with that of the *lyl1-1* mutant (Wang et al., 2014). Moreover, it was revealed that an *O. sativa* mutant containing an inactivated *CHLP* gene could still produce tocopherols (Kimura et al., 2018). *CHLP* also plays an important role in the abiotic stress response. In tomatoes, *CHLP* overexpression improves seedling growth and tolerance to salt, osmotic, and oxidative stress by regulating the synthesis of chlorophyll a (Chl a) and tocopherol (TP) (Liu et al., 2015). However, the transcriptional regulatory mechanism of *CHLP* in plants has rarely been reported.

Recently, woody plants (such as *Populus*) have been proposed as models for the study of gene function due to their fast-growing properties and clear genomic information (Tuskan et al., 2006; He et al., 2021b,c). However, the role of the poplar *CHLP* family in plant growth and development has not been reported. Three putative CHLP proteins have been identified in the whole genome of *P. trichocarpa*. Comparative genomics, transcriptomics, and RT-qPCR were performed to investigate the poplar *CHLP* family and lay the foundation for studies on the features and functions of *CHLPs* in poplar growth and development. Moreover, inhibition of *PtrCHLP3* expression in poplar can inhibit plant growth through the downregulation of photosynthesis. Furthermore, we investigated the role of *PtrCHLP3* in the molecular regulatory networks of photosynthesis and growth. Therefore, this study provides genetic resources and a molecular basis for improved breeding of fast-growing tree traits.

## Materials and Methods

### Identification and Phylogenetic Analysis of the *CHLP* Gene Family

The identification and analysis of the *CHLP* gene family were based on the whole genome of *P. trichocarpa.* The relevant *Populus* genomic and protein sequences were downloaded from the Phytozome13 database^1^ (Goodstein et al., 2011). The other genomic data and protein sequences of *Arabidopsis thaliana, Gossypium raimondii, Micromonas pusilla, Chlamydomonas reinhardtii, Triticum aestivum, Prunus persica, Volvox carteri, Oryza sativa, Arabidopsis lyrata, Trifolium pratense, Daucus carota, Phaseolus vulgaris, Populus deltoides, Sorghum bicoloa, Chromochloris zofingiensis, Lactuca sativa, Zea mays, Miscanthus sinensis, Setaria viridis, Botryococcus braunii, Panicum virgatum, Salix purpurea, Gossypium hirsutum, Dioscorea alata, Mimulus guttatus, Thuja plicata, Schrenkiella parvula, Spinacia oleracea, Phaseolus acutifolius, Diptychocarpus strictus, Cleome violacea, Panicum hallii, Zostera marina, Manihot esculenta, Paspalum vaginatum, Fragaria vesca, Glycine max, Solanum tuberosum, Quercus rubra, Solanum lycopersicum*, and *Hordeum vulgare* were also downloaded from the Phytozome13 database. We used conserved domains GG-red-SF (TIGR02032) as seed sequence to search *CHLP* genes from the above species, the correct *CHLP* family genes were obtained using the CDD^2^ database (Lu et al., 2019).

To perform a more integral analysis of the *CHLP* gene family, all identified *CHLP* protein sequences were combined and compared using Clustal W, and a genealogical tree was constructed based on the neighbor-joining (NJ method, 1000 bootstraps) in MEGA-X (Hall, 2013). The results of the phylogenetic tree were drawn and modified using FigTree software (v1.4.3). Each CHLP protein was named according to the order in which it was located on a chromosome.

### Structure, Conserved Motif, Gene Duplication, *Cis*-Element, and Expression Patterns Analysis of *PtrCHLPs*

All bioinformatics analyses of the *CHLPs* were performed as previously described (He et al., 2021a,b). Structure and conserved motif analysis of the *CHLP* gene family was performed using the Gene Structure Display Server (GSDS^3^) (Hu et al., 2014) and Multiple Em for Motif Elicitation (MEME^4^) online software (Bailey et al., 2015). Gene collinearity analysis of *CHLP* genes within and between species were performed using the Multiple Collinearity Scan toolkit (MCScanX). Moreover, *cis*-element analysis of *PtrCHLPs* were performed using the Plant *Cis*-Acting Regulatory Element (PlantCARE^5^) database (Lescot et al., 2002), and corresponding organization expression data was acquired from the Popgenie^6^ database (Sjodin et al., 2009), then graphics visualization was performed using Tbtools (Chen et al., 2020).

### RNA Extraction and RT-qPCR Analysis

The RNA of 84 K poplar (*P. alba* × *P. glandulosa)* leaves, stems, roots, buds, phloem, and xylem was extracted using a plant RNA extraction kit (Accurate Biology, Hunan, China) and converted into cDNA using a reverse transcription kit (Accurate Biology, Hunan, China). Primers were designed by Premier 5.0 and synthesized by TSINGKE Biology Co., Ltd. (Chengdu, China). *PtrUBQ* and *PtrActin* were used as internal reference genes, and cDNA was used as a template for RT-qPCR amplification (He et al., 2018, 2019). Five biological replicates and four technical replicates were performed for each sample. PCR was performed on a Bio-Rad CFX96 instrument (Bio-Rad, Hercules, United States) and the 2^–ΔΔ^ ^Ct^ algorithm was used to analyze the results (Livak and Schmittgen, 2001).

### Genetic Transformation of Poplar

First, we cloned *PtrCHLP3* from *P. trichocarpa*, which was subsequently cloned into the pEASY-T1 Kit vector and sequenced. The *PtrCHLP3* fragment was amplified from the positive construct *PtrCHLP3*- pEASY. Double enzyme digestion (*Sac*I/*Xba*I) was used to retrieve the pCambia2301-PS vector. The forward sequences of partial fragments of *PtrCHLP3*, sequences of RTM, and reverse sequences of partial fragments of *PtrCHLP3* were sequentially recombined into the PCAMbiA2301-PS vector using the ClonExpress^®^ II One Step Cloning Kit (Vazyme Biotech Co., Ltd, Nanjing, China). The correct clone, pCAMBIA2301-*PtrCHLP3*–RNAi, was introduced into *Agrobacterium tumefaciens* strain GV3101. The RNAi expression vector was transferred into poplar 84 K using *Agrobacteria*-mediated transformation methods (He et al., 2019). Transgenic poplar 84 K seedlings were selected in woody plant medium (WPM) containing 60 mg/L kanamycin, and the transformants were confirmed by PCR and stained with β-glucuronidase (GUS). Transgenic poplar lines were obtained and used in subsequent experiments. Related primers (Supplementary Table S1) were designed using Primer- Primer 6.0 tools.

### Histochemical Staining of β-Glucuronidase

β-Glucuronidase tissue staining of the plants was performed as previously described (Yang et al., 2020). Histochemical staining of the leaves was performed using the M5 HiPer GUS staining kit (Mei5bio, Beijing, China). The prepared leaves were soaked in GUS dye solution overnight. The leaves were then treated with 70% ethanol 2–3 times until the negative control material was white.

### Plant Materials and Growth Conditions

Wild type (WT) and transgenic poplar lines (chlp-3 and chlp-4) were the present study. Thirty-day-old plantlets were transplanted to pots and grown in a greenhouse (20–25°C; 70% humidity; 16 h light, 8 h dark) at Wenjiang, Chengdu, China (30°70’N, 103°85’E, 537.11 m above sea level) (He et al., 2020). All plants were cultivated in the same-sized pots with trays. After the plants grew normally in the greenhouse for 1 month, follow-up experiments were conducted.

### Physiological and Biochemical Analysis

The plant height and stem elongation rate between WT and transgenic poplar were measured every 7 days, and the fresh weight and ground diameter of the plants were measured after 35 days of harvest. Measurement and calculation methods of plant height and stem, and stem elongation rates were based on published papers (Wang et al., 2016; He et al., 2018). Each sample was assayed in ten biological replicates. The ninth to eleventh leaves of the WT and transgenic poplar were measured using the LI-6800 Portable Photosynthesis System (LI-COR companies, Lincoln, United States). The light intensity and CO_2_ concentration were set as 800 μmol m^–2^ s^–1^ and 400 μmol mol^–1^, respectively, in the leaf chamber to measure the leaf net photosynthetic rate (Pn), transpiration rate (Tr), stomatal conductance (Gs), and instantaneous water use efficiency (WUE, Pn/Tr). Photosynthetic light response curves were determined at photosynthetically active radiation (PAR) levels of 1600, 1200, 1000, 800, 600, 400, 200, 100, 75, 50, 25, and 0 μmol m^–2^ s^–1^ to measure Pn (He et al., 2018). In addition, a non-rectangular hyperbolic model was used to calculate the Pn–light curve and obtain the initial quantum yield and Pmax (Thornley, 1976).

Chlorophyll content was detected in the terminal bud and leaves 1–8 (from top to bottom) of poplars using a portable chlorophyll meter (SPAD-502Plus, Konica Minolta, Osaka, Japan). Chlorophyll and carotenoids were extracted from fresh leaves using 80% acetone. The total chlorophyll (Chl), chlorophyll a (Chl a), chlorophyll b (Chl b), and carotenoid contents were measured using a spectrophotometer (UV-3800, Unico, Shanghai, China) and quantified according to the methods described by Arnon (1949). The parameters of the slow dynamic fluorescence curves, including the maximal PSII quantum yield (Fv/Fm), quantum yield of photochemical energy conversion in PSII [Y(II]), photochemical quenching parameter (qP), and non-photochemical quenching parameter (NPQ), were measured as previously described (He et al., 2018). Each parameter was measured in at least 40 replicates, including four technical repeats and ten biological repeats.

### Tissue Section and Staining Analysis

Toluidine blue and phloroglucinol-HCl staining were performed as previously described (Gui et al., 2019), with slight modifications. Toluidine blue and phloroglucinol-HCl were used only to show the cell profile and the distribution and size of tissues in various parts of the poplar stem. The mature leaves of WT and transgenic poplars were located near the 8th internode. Transverse sections of the 8th internode of the WT and transgenic poplar were obtained using a semi-automatic frozen slicer. The cells were stained with 10% toluidine blue stain and 1% phloroglucinol (w/v) in 25% HCl and observed under a microscope (Olympus BX51, Tokyo, Japan), respectively. In addition, the number of cell layers and the proportion of xylem and bark in the cell cross-section were counted. Each parameter was repeated at least a hundred and twenty times, including three technical and 40 biological repeats.

### Bioinformatics Analysis of Possible Upstream Transcription Factors and Co-expressed Gene of *PtrCHLPs*

The possible upstream transcription factors (PUTFs) for *PtrCHLP3* were identified using the Plant Transcriptional Regulatory Map database (PlantRegMap^7^) (Tian et al., 2019). Moreover, the tissue expression of PUTF genes was obtained from the Popgenie database, and the co-expressed gene expression of *PtrCHLP3* was obtained from Phytozome13 databases. Heat map expression patterns were drawn using TBTools (Chen et al., 2020).

### Statistical Analyses

Microsoft Excel 2019 was used to calculate the average and standard error of all data. Moreover, Student’s *t*-test in SPSS software (v26.0) was applied to analyze the significance of the differences between control and treatment groups, and *P*-values were used to determine significant differences (*P < 0.05; ^**^P < 0.01). Duncan’s multiple comparisons *post hoc* test was used to determine *P*-values among different poplar tissues. All the data were normalized and conformed to a normal distribution.

## Results

### Analysis of Phylogenetic Tree, Gene Structure, Protein Motif, and Conservative Domain of the *CHLP* Family

**FIGURE 1:**
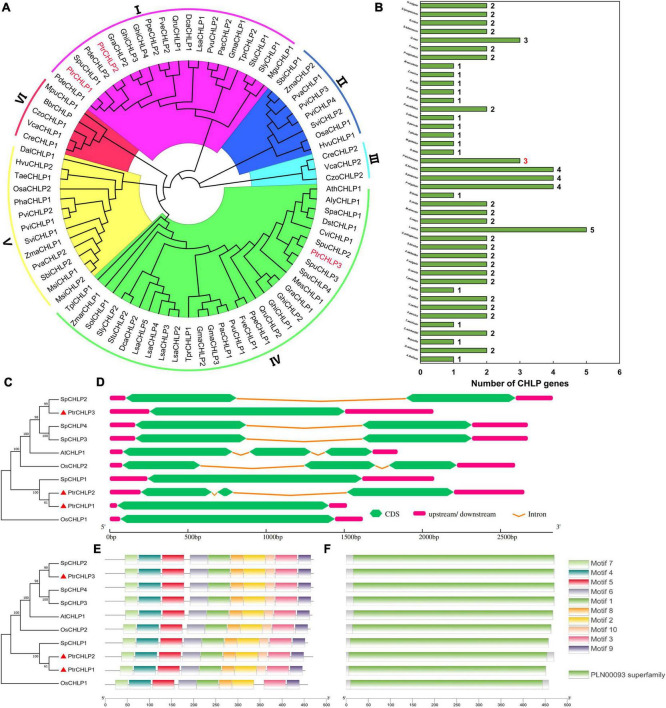
The phylogenetic tree and molecular structure analyses of *PtrCHLP*. **(A)** Evolutionary and phylogenetic analysis of the *CHLP* family among different plants. **(B)** Comparisons of *CHLP* protein numbers across 42 plant species. The accession numbers and gene names are all shown in Supplementary Table S2. The phylogenetic tree **(C)**, gene structure **(D)**, conserved motif **(E)**, and conserved protein structure **(F)** analyses of *CHLP* genes from *A. thaliana*, *O. sativa*, and *P. trichocarpa*. The length of each model is exhibited in proportion.

The 82 amino acid sequences of CHLP from 42 plants were used to establish a biogenetic tree to further investigate the evolution and discrepancy of CHLP family proteins between poplar and other plants (Figure 1A). All *CHLP* genes were divided into six groups (I–VI), and three *CHLP* genes were identified in poplar. *PtrCHLP* proteins were found in groups I and IV. The phylogenetic tree indicated that the *CHLP* genes of *P. trichocarpa* and *S. purpurea* were close to each other, indicating high homology. Statistical analysis of the *CHLP* genes in 42 plant species showed that 85.7% of the plants had no more than two *CHLP* genes (Figure 1B and Supplementary Table S2). Surprisingly, only one *CHLP* gene was found in the model plant *Arabidopsis*, whereas a maximum of five *CHLP* genes were found in *L. sativa*. Generally, the *CHLP* gene family differentiates and multiplies slowly during plant evolution.

To further analyze the phylogenetic relationships among the diverse members of the CHLP family, an evolutionary tree was constructed based on ten CHLP proteins from *A. thaliana*, *O. sativa*, *S. purpurea*, and *P. trichocarpa* (Figure 1C); and their gene structures, motifs, domains, and transmembrane domains were predicted. The phylogenetic tree showed that *PtrCHLP1* and *PtrCHLP2* clustered on the same branch, but were far from *PtrCHLP3*, suggesting that there may be functional differentiation between *PtrCHLP1/2* and *PtrCHLP3.* In addition, *PtrCHLP3*, *SpCHLP2/3/4*, *OsCHLP2*, and *AtCHLP1* were clustered in the same clade, suggesting that they may have similar biological functions. Most *CHLP* genes had one or two introns, whereas *PtrCHLP1/3*, *SpCHLP1*, and *OsCHLP1* had no introns (Figure 1D). Moreover, one geranylgeranyl diphosphate reductase domain (PLAN00093 Superfamily) was discovered in the PtrCHLP protein (Figure 1F and Supplementary Table S3) and was made up of motif (1–10) components (Figure 1E and Supplementary Table S4). However, the C-terminus of OsCHLP1 lacks Motif10. The CHLP family is highly evolutionarily conserved.

### Analysis of Chromosomal Location and Gene Duplication

**FIGURE 2:**
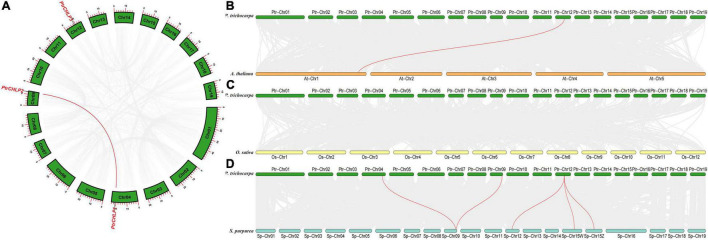
Synteny analysis of poplar *CHLP* gene family. **(A)** The synteny analysis of *CHLP* genes in *P. trichocarpa*. **(B–D)** Synteny analysis of *CHLP* genes between poplar and the other three typical plant species (*A. thaliana, O. sativa*, and *S. purpurea*). The synteny blocks in poplar and the other species are shown in gray lines, whereas the collinearity of *CHLP* gene pairs is emphasized in red lines from different species.

The chromosomal position, collinearity, and evolution of *CHLP* genes in poplar were investigated to explore the phylogenetic relationship between *CHLP* family genes. The *PtrCHLPs* gene was located on chromosomes 4, 9, and 12 (Figure 2A and Supplementary Table S5). Chromosome regions of less than 200 kb of two or more genes were deemed tandem doubling events (Xie et al., 2018). The *PtrCHLP* gene family does not form tandem repeat regions on poplar chromosomes. However, two genes were represented by complex segmental duplication events (*PtrCHLP1/2*) using the MCScanX method (Singh et al., 2017). In summary, duplicated gene pairs (*PtrCHLP1/2*) were highly homologous, implying that they may jointly regulate the same biological pathways.

We compared collinear graphs among *P. trichocarpa, A. thaliana, O. sativa*, and *S. purpurea* to detect the evolutionary relationship of the *CHLP* gene in these species (Figures 2B–D). The collinear graphs showed that five pairs of isogenous genes were found between poplar and willow, and one pair of isogenous genes (*PtrCHLP3* vs. *AtCHLP1*) was identified between poplar and *A. thaliana* (Figure 2B and Supplementary Table S5). The *PtrCHLP3* genes were found to have a relationship with three isogenous gene (*SpCHLP2/3/4*) pairs in willow, suggesting that this gene has undergone a lot of differentiation in willows. Nevertheless, there are no *CHLP* homologous genes between poplar and rice, implying that many *CHLP* genes may be newly differentiated in dicotyledons (Figure 2C).

### *Cis*-Element Analysis and Expression Patterns of *PtrCHLPs* in Poplar

**FIGURE 3:**
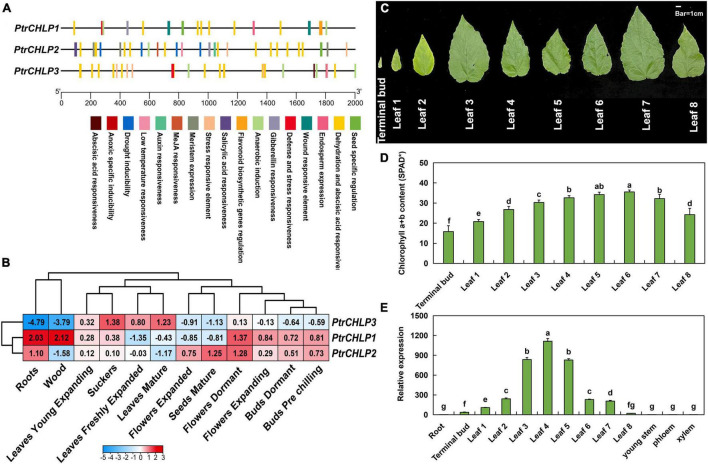
Analysis of *cis*-acting regulatory elements and expression patterns of CHLPs in *Populus*. **(A)** Analysis of *cis*-acting regulatory elements of *PtrCHLPs*. The corresponding positions of *cis*-elements correlated with stress and growth in *PtrCHLP* promoter regions. Diverse colors indicate various *cis*-acting elements, and their regions conform to the homologous region of the promoter. **(B)** Heat maps represent the expression of *PtrCHLP1-3* in different plant tissues at various growth stages. The color bars indicate the range of fold change in expression. The phenotype **(C)** and chlorophyll content **(D)** analysis of 30-day-old poplar leaves from top to base. **(E)** RT-qPCR analysis of the expression features of *PtrCHLPs* in different tissues. The bars indicate mean ± SD (n = 20) from five independent trials. Different letters above the bars indicate statistically significant differences (adjusted *P* < 0.05, one-way ANOVA).

The upstream sequences (∼2,000 bp) of the promoter were obtained from the genomic sequence of poplar to confirm the expression features of *PtrCHLP1/2/3*. The PlantCARE service was explored for *cis*-elements in the *PtrCHLP* promoter (Figure 3A). The detailed effects of these motifs (*cis*-elements) are listed in Supplementary Table S6. These elements are involved in plant growth and development, phytohormone responsiveness, and abiotic and biotic stress responses. All kinds of *cis*-acting elements related to development were explored, including the auxin responsiveness element, endosperm expression, gibberellin responsiveness element, and seed-specific regulation, all of which indicated the crucial role of *cis*-acting elements in poplar development of *CHLP.*

To further study the function of *CHLP* genes in growth and development, we investigated the tissue expression profiles of *PtrCHLP1/2/3* in transcriptome data (Figure 3B and Supplementary Table S7). *PtrCHLP1/2* showed highly similar expression patterns in tissues other than wood, expanded flowers, and mature seeds. However, the expression pattern of *PtrCHLP3* was the opposite to that of *PtrCHLP1/2*, which was upregulated in photosynthetic organs (leaves) but downregulated in non-photosynthetic organs, especially in roots and xylem. Therefore, we hypothesized that *PtrCHLP3* might be involved in chlorophyll biosynthesis in poplars. To better determine the function of these genes, we compared the absolute expression levels of three *PtrCHLP1/2/3* genes in different tissues. The results showed that the absolute expression level of *PtrCHLP3* was the highest in mature leaves, and the tissue expression model was similar to the relative expression pattern (Supplementary Figure S3).

One-month-old poplar leaves were analyzed from top to bottom to determine the phenotype and chlorophyll content. From top to bottom, the leaves changed color from pale green to green, and then to yellow (Figure 3C). Similarly, chlorophyll content first increased and then decreased, reaching the highest content in the sixth leaf (Figure 3D). Furthermore, the tissue expression pattern of poplar was analyzed using RT-qPCR (Figure 3E), and the relative expression of *PtrCHLP3* in the photosynthetic organs (buds and leaves) of poplar was relatively high. In contrast, its expression levels were lower in the non-photosynthetic tissues. Overall, this tissue-specific expression suggests that *PtrCHLP3* may be involved in chlorophyll biosynthesis in poplars.

### Acquisition and Phenotypic Analysis of Transgenic Poplar

**FIGURE 4:**
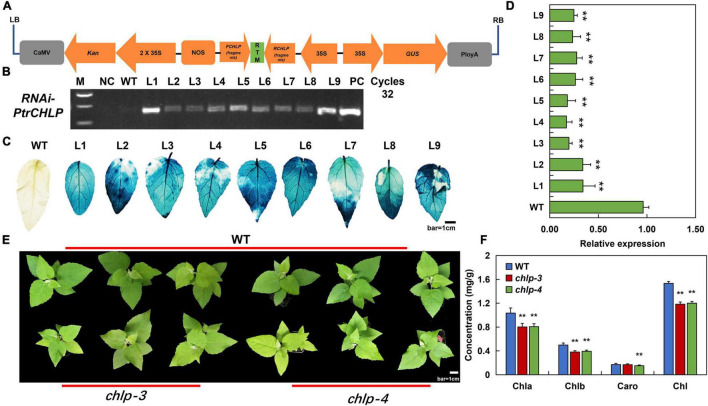
Analysis of the transgenic poplar plants suppressing *PtrCHLP3*. **(A)** Diagrammatic drawing of pCAMBIA2301-*PtrCHLP3*–RNAi vector. Expression of *dsRNA-PtrCHLP3* is activated by the double 35S promoter. **(B)** PCR analysis of transgenic poplar: M, DNA molecular weight marker; PC, p2301-*PtrCHLP*–RNAi positive control vector; WT, wild-type; NC, negative control; L1-9, PCR products with genomic DNA from regenerated kanamycin-resistant poplar 84 K leaves as a template. **(C)** Histochemical GUS staining of different transgenic lines. Bar = 1 cm. **(D)** RT-qPCR analysis of *PtrCHLP3* expression levels in different transgenic lines (*chlp1–9*). The phenotype **(E)** and chlorophyll content **(F)** of WT and *PtrCHLP3*–RNAi lines (*chlp3 and chlp4*). Bar = 1 cm. The value represents the mean ± standard deviation (*n* = 20). Asterisks indicate a significant difference at the same level: ** indicates *P* < 0.01.

To investigate whether *PtrCHLP3* affects chlorophyll biosynthesis in plants, the correct recombinant *pCAMBIA2301-PtrCHLP3–RNAi* vector was inserted into the poplar 84 K genome (Figure 4A). At least nine independently regenerated kanamycin-resistant lines were acquired, and nine transgenic lines (L1–L9) were detected using PCR analysis. We detected the predicted 500 bp band in the nine transgenic lines (Figure 4B). Furthermore, transgenic poplars were verified by GUS staining (Figure 4C). The relative expression levels of *PtrCHLP3* in these transgenic lines were significantly lower than those in the WT, indicating that the *pCAMBIA2301-PtrCHLP3–RNAi* vector inhibited the expression of *PtrCHLP3* in poplar 84 K. Because the transcript abundance of two independent transgenic lines (L3 and L4) was lower than that of the other lines (Figure 4D), *chlp3* and *chlp4* were selected for subsequent experiments.

In addition, phenotypic analysis showed that the leaf color of *chlp3* and *chlp4* was significantly yellow compared to that of the WT (Figure 4E). Meanwhile, chlorophyll content analysis indicated that the contents of chlorophyll a, chlorophyll b, and total chlorophyll in the leaves of transgenic plants were significantly lower than those in the leaves of WT (Figure 4F). In summary, the suppression of *PtrCHLP3* expression may lead to the downregulation of chlorophyll levels in plants

### *PtrCHLP3* Involved in Poplar Growth

**FIGURE 5:**
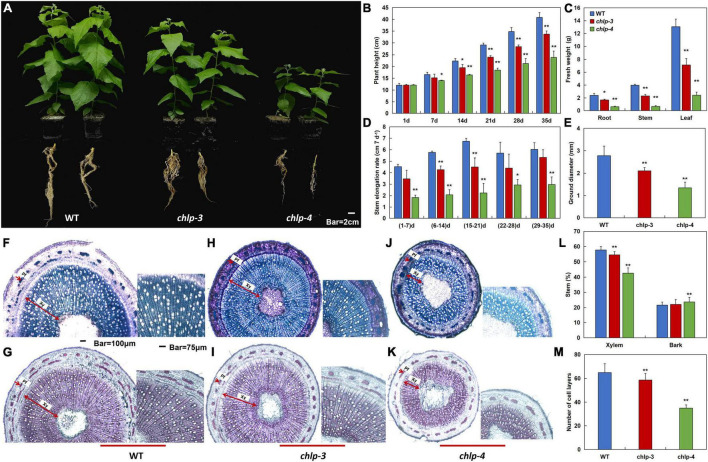
*PtrCHLP3* positively regulates poplar growth. **(A)** Scrubby appearance of 35-day-old plants of independent PtrCHLP3-RNAi transgenic lines (*chlp3* and *4*). **(B–E)** The values of plant height **(B)**, fresh weight **(C)**, stem elongation rate **(D)**, and stem diameter **(E)** of WT and *chlp* plants. **(F–K)** Transection and staining via toluidine blue **(F,H,J)** and phloroglucinol-HCl **(G,I,K)** of the 8th internode of WT and *chlp* plants. Pf, phloem fibers; Xy, xylem. **(L)** Secondary xylem (%) and bark (%) in stem between WT and *chlp* poplars. The scope of secondary xylem, bark, and total stem was counted using ImageJ in toluidine blue- and phloroglucinol-stained anatomical segments of the 8th internode of WT and *chlp* poplars. **(M)** Quantification of secondary xylem cell layers in WT and *chlp* poplars. The amount of secondary xylem cell layers was calculated in toluidine blue- and phloroglucinol-stained anatomical segments of the 8th internode of WT and *chlp* plants. The value represents the mean ± standard deviation (*n* = 120). Asterisks indicate a significant difference at the same level: ** indicates *P* < 0.01.

To further study the effects of *PtrCHLP3* on the growth and development of poplar, WT and transgenic poplars were observed for 35 days under normal conditions. Phenotypic analysis showed that the height of the aboveground part and root length of transgenic poplar were significantly inhibited compared with those of WT (Figure 5A). With the time of growth, the plant height and stem elongation rate of WT plants were significantly higher than those of the transgenic plants (Figures 5B,D). Furthermore, the fresh weights of the transgenic poplar roots, stems, and leaves were significantly lower than those of the WT, and correspondingly decreased by 30–75%, 42–84%, and 46–82%, respectively (Figure 5C). In contrast to the WT, the stem diameter of the transgenic plants was significantly smaller (Figure 5E).

To further understand how *PtrCHLP3* affects the thickness of poplar stems, toluidine blue and phloroglucinol-HCl were applied to dye a cross-section of the 8th internode of WT and *chlp* plants (Figures 5F–K). Phloroglucinol-HCl staining was performed to visualize lignin in the cell walls (red violet). In wild-type and transgenic plants, we observed no difference in the deposition of lignin in xylem, phloem fiber, and pith cells (Figures 5G,I,K). The widths of the phloem and xylem of the transgenic poplar were significantly thinner than those of the WT. In addition, compared to WT, the proportion of transgenic xylem in the stems was significantly reduced (Figure 5L). However, there was no significant difference in the bark ratio between transgenic and WT stems. Consistent with previous results, quantification of secondary xylem cell layers showed that xylem cell layers of transgenic plants were significantly smaller than those of WT plants (Figure 5M). These results suggest that the suppression of *PtrCHLP3* expression can inhibit xylem expansion in poplars. In conclusion, *PtrCHLP3* plays an important role in regulating poplar growth.

### *PtrCHLP3* Actively Regulates Photosynthesis in Poplar

**FIGURE 6:**
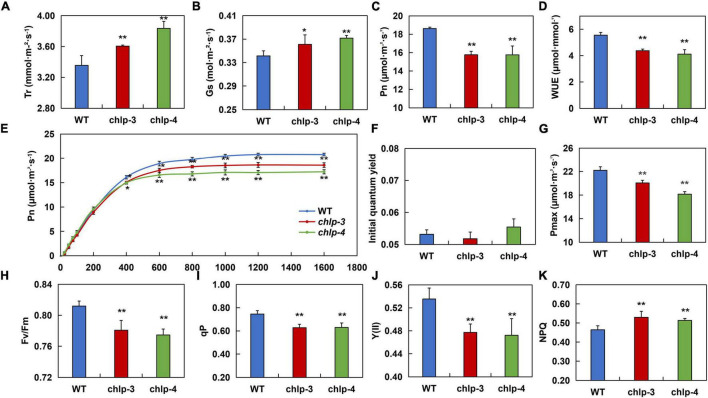
*PtrCHLP3* actively regulates photosynthesis. **(A–D)** The photosynthetic parameters of *chlp* plants relative to those of WT plants. Tr, transpiration rate; Gs, stomatal conductance; Pn, net photosynthetic rate; WUE, instantaneous water-use efficiency; **(E–G)** Pn–light curve, initial quantum yield, and Pmax were measured in WT and *chlp* plants. **(H–K)** The value [Fv/Fm, Y (II), qP, and NPQ] of the slow dynamic fluorescence induction curve were measured in *chlp* and WT plants. The value represents the mean ± standard deviation (*n* = 40). Asterisks indicate a significant difference at the same level: * indicates *P* < 0.05; ** indicates *P* < 0.01, respectively.

To thoroughly study how *PtrCHLP3* promotes plant growth, the photosynthetic index (Pn, Gs, Tr, and WUE) was compared between WT and transgenic poplars. The result showed that the Tr and Gs in transgenic plants were significantly larger than those in WT poplar (Figures 6A,B). However, the net photosynthesis (Pn) and water use efficiency (WUE) of transgenic plants were significantly lower than those of the WT plants (Figures 6C,D). Through simulation of photosynthesis-light response curves of transgenic and WT 84 K poplar, we found that when the light intensity was higher than 400 μmol m^–2^ s^–1^, the Pn of the transgenic plants was significantly lower than that of the WT (Figure 6E). However, there was no significant difference in the initial quantum yield between WT and transgenic poplars (Figure 6F). Among them, the Pmax of wild-type poplar 84 K was 22.202 μmol m^–2^ s^–1^, and those of the transgenic plants *chlp3* and *chlp4* were 20.064 μmol m^–2^ s^–1^ and 18.165 μmol m^–2^ s^–1^, respectively (Figure 6G). These results suggest that *PtrCHLP3* influences the photosynthetic rate of plants through non-stomatal factors.

To thoroughly determine how *PtrCHLP3* affects electron transport during photosynthesis, fluorescence parameters [Fv/Fm, Y (II), qP, and NPQ] were measured between transgenic and WT plants. The results showed that the maximal Fv/Fm, qP, and Y (II) of transgenic plants were lower than those of WT plants (Figures 6H–J). In addition, NPQ was significantly higher in the transgenic plants than in WT (Figure 6K). In general, the downregulation of *PtrCHLP3* levels in plants reduces electron transport rates during photosynthesis, thus weakening photosynthesis.

### Molecular Network of *PtrCHLP3* in Regulating Photosynthesis and Chlorophyll Biosynthesis in Poplar

**FIGURE 7:**
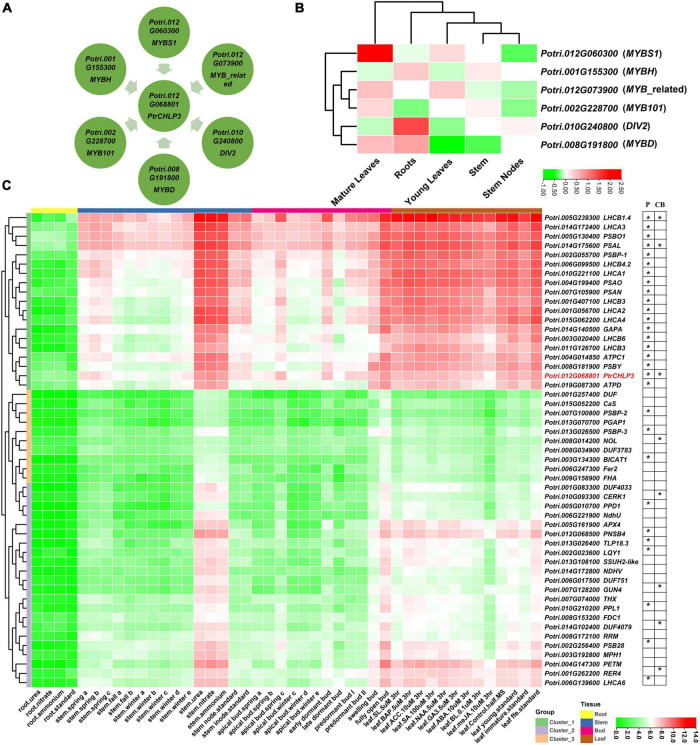
Construction of PtrCHLP3 molecular regulatory network. Bioinformatic analysis of TFs of *PtrCHLP3*. **(A)** Interaction network analysis of TFs-*PtrCAX3*. The blue rectangle represents TFs upstream of *PtrCHLP3*. **(B)** Heat maps showing the transcriptional abundance of upstream transcription factors of *PtrCHLP3* under different plant tissues. The range of fold change in expression in the heat map is indicated by the color bar. **(C)** Cluster heat map analysis of genes co-expressed with *PtrCHLP3* in different plant tissues and treatments. P, Photosynthesis; CB, Chlorophyll biosynthesis. The asterisk indicates that the gene is involved in the process of P or CB.

To further elucidate the molecular mechanism by which PtrCHLP3 regulates photosynthesis and growth of poplar, the potential upstream transcription factors (PUTFs) of *PtrCHLP3* were analyzed. The PUTFs of *PtrCHLP3* were identified through bioinformatics, and their functions and expression levels in various tissues were analyzed (Figure 7B and Supplementary Table S9). Surprisingly, the PUTFs (Potri.012G060300, Potri.012G073900, Potri.010G240800, Potri.008G191800, Potri.002G228700, and Potri.001G155300) of *PtrCHLP3* are MYB TF (Figure 7A and Supplementary Table S8). Potri.012G060300, Potri.012G073900, and Potri.002G228700 had similar expression patterns to *PtrCHLP3*, which were upregulated in mature leaves but inhibited in roots and stems. In contrast to the expression pattern of *PtrCHLP3*, Potri.010G240800 and Potri.001G155300 were downregulated in leaves but activated in roots and stems. In addition, the expression pattern of Potri.008G191800 is complex, and it is upregulated in both roots and mature leaves but downregulated in young leaves and stems.

In addition, we constructed cluster heat maps co-expressed with *PtrCHLP3* in different tissues (Figure 7C), which could be divided into three clusters (1–3). All genes in Cluster 1 were downregulated in roots, most were upregulated in stems and buds, and all were significantly upregulated in leaves. However, the gene expression level of Cluster 2 in each poplar tissue was almost suppressed. Furthermore, all genes in Cluster 3 were inhibited in roots, most were downregulated in stems and buds, and some were activated in leaves. A large number of genes involved in photosynthesis and chlorophyll biosynthesis were found to form a co-expression network with *PtrCHLP3*. Moreover, the following genes with similar expression patterns to *PtrCHLP3* are located in Cluster 1: LIGHT HARVESTING COMPLEX PHOTOSYSTEM II SUBUNIT (*LHCB1.4/3/4.2/4/6*), PHOTOSYSTEM I LIGHT HARVESTING COMPLEX GENE (*LHCA1/2/3/4*), PHOTOSYSTEM II SUBUNIT O *(PSBO1)*, PHOTOSYSTEM I SUBUNIT L *(PSAL)*, PHOTOSYSTEM II SUBUNIT P *(PSBP1)*, PHOTOSYSTEM I SUBUNIT O *(PSAO)*, GLYCERALDEHYDE 3-PHOSPHATE DEHYDROGENASE A SUBUNIT *(GAPA), ATPC1*, ATP synthase D chain *(ATPD), PSAN*, and PHOTOSYSTEM II BY *(PSBY*); and they are all involved in plant photosynthesis. In addition, 25.755% of the genes were found to play a role in chloroplasts by GO cell component analysis. At the same time, GO biological process analysis found 14.085% genes involved in plant photosynthesis (Supplementary Figure S2). In conclusion, *PtrCHLP3* and its co-expressed genes play important roles in regulating photosynthesis and chlorophyll biosynthesis in poplars.

## Discussion

Chlorophyll is the most abundant pigment in photosynthetic organs and plays an irreplaceable role in chloroplast formation, homeostasis, and photosynthesis (Lin et al., 2014; Zhang et al., 2021). The final stage of Chl a synthesis is the esterification of the tetrapyrrole moiety and chlorophyllide. CHLP, a key enzyme in the final step of Chl a biosynthesis, participates in the terminal hydrogenation step of chlorophyll synthesis (Addlesee and Hunter, 2002). The CHLP family has been defined and explored in many plants, such as *Arabidopsis*, ralgae, tobacco, rice, peach, olive, and tomato (Addlesee and Hunter, 2002; Shpilyov et al., 2005; Deng et al., 2014; Zhang et al., 2015; Tan et al., 2019). However, there has been no related work on its distribution and function in poplar. We used comparative genomics, transcriptomics, and molecular biology to identify *CHLP* family genes in poplars and analyze their effects on plant photosynthesis and growth.

### Phylogenesis, Molecular Characteristics, and Expansion of the *CHLP* Gene Family

We established a genealogical tree using the protein sequences of 42 plants and divided them into six groups to explore the evolution and phylogeny of *CHLP* genes (Figure 1A). *PtrCHLP1*, *PtrCHLP2*, and *PtrCHLP3* were located on different branches (Figures 1A,C), indicating that *CHLP* genes may be functionally differentiated in poplar trees. Macromolecules (genes or proteins) with similar gene and protein structures may have similar biological functions (He et al., 2021b). Moreover, *AtCHLP1* and *PtrCHLP3* have similar conserved domains and motifs, and their clustering on the same branch indicates that they may be used for similar biological functions. However, motif10 was absent from the C-terminus of *OsCHLP1* (Figure 1C), resulting in a single branch (Figure 1E).

In addition, the phylogenetic analysis of family genes can explain the evolution of genes (Zheng et al., 2021). *Salix* and poplar are closer because they belong to the same genus (Xiao et al., 2018; He et al., 2021a). Poplar and *Salix* are relatively tall dicotyledonous trees, and their *CHLP1* genes clustered together (Figures 1A,C). Tandem and segmental replication play critical roles in the evolution of species (Kaur et al., 2017; Xie et al., 2018). One pair of genes (*PtrCHLP1/2*) that appeared as segmental duplications in *Populus* suggested that gene duplications played a role in the evolution of *PtrCHLP* genes in the genus (Figure 2A). Similar to the results of the evolutionary trees, only gene pairs formed between *AtCHLP1* and *PtrCHLP3* in *Arabidopsis* and poplar (Figure 2B), which ensured that their functions were similar and conserved. However, many gene pairs were detected between *Populus* and *S. purpurea* (Figure 2D), implying that conspicuous variation and duplication of *CHLP* genes could have been generated during the evolution of dicotyledons of the same genus.

### Expression Pattern Analysis of *CHLP* in Poplar

The level of gene expression is very important for plant growth, development, and external responses (Khatun et al., 2017; He et al., 2021c). With the differentiation and amplification of genes, the expression profiles of different members of a gene family vary greatly (Mao et al., 2021). According to the transcriptomic data of poplars, the expression of *PtrCHLP1/2/3* fluctuated slightly in each poplar tissue (Figure 3B). In addition, the tissue expression patterns of *PtrCHLP1/2* and *PtrCHLP3* were the opposite, which may be closely related to cis-acting elements on the gene promoter (Figure 3A). The *cis*-acting elements of promoters play an important role in the regulation of gene transcription (He et al., 2019). The promoter of *PtrCHLP1/2* mainly contains elements that respond to hormones and stress, whereas the promoter of *PtrCHLP3* contains many elements that are correlated with growth and development compared with *PtrCHLP1/2* (Figure 3A and Supplementary Figure S1). *CHLP* has been reported to play a critical role in chlorophyll synthesis and is mainly expressed in the leaves (Shpilyov et al., 2005; Zhang et al., 2015). Consistent with the above results, *PtrCHLP3* was significantly upregulated in leaves but inhibited in non-photosynthetic organs (Figure 3B). In addition, the expression levels of *PtrCHLP3* in different leaves were different (Figure 3E). Although *CHLP* is involved in the last step of chlorophyll a synthesis, its expression level in leaves is not completely synchronous with chlorophyll content (Figures 3D,E). Mature leaves play a leading role in the photosynthetic contribution of plants, whereas young leaves need to synthesize a large amount of chlorophyll in the process of growing to mature leaves (Luo et al., 2019; Shen et al., 2021). This may be the main reason the expression of *PtrCHLP3* reaches its peak before chlorophyll content in leaves; after all, the response rate of genes is faster than the phenotype.

### *PtrCHLP3* Is Involved in Plant Growth and Development Through Photosynthesis

In the present study, gene-silenced transgenic poplars (*RNAi-PtrCHLP3/chlp3 and chlp4*) were obtained and used for subsequent functional verification experiments (Figure 4). Compared with WT plants, the leaves of gene-silenced poplar plants were yellow due to the inhibition of chlorophyll synthesis (Figures 4E,F). Consistent with previous results, downregulation of *PtrCHLP3* resulted in decreased chlorophyll content in plants (Figure 4D). In antisense *N. tabacum* transformants of *CHL I*, chlorophyll content in leaves was lower, and the leaf phenotype was light green or with white and green patches (Jutta Papenbrock et al., 2000; Havaux et al., 2003). The inhibition or reduction of chlorophyll synthesis results in the inhibition of plant growth and development (An et al., 2020; He et al., 2020). The plant height, stem diameter, and biomass of *chlp3/4* were significantly lower than those of WT poplars (Figures 5A–E), which may be caused by severe chlorosis of the plant. It has been reported that GATA deletion in poplars inhibits chlorophyll synthesis, leading to yellowing and biomass reduction (An et al., 2020). This obstruction of chlorophyll synthesis may also be caused by variations in the chloroplast structure (An et al., 2020). However, the decrease in chlorophyll synthesis in *chlp3/4* was mainly attributed to downregulation of the geranylgeraniol reductase (*CHLP*) gene (Figures 4D,F).

However, this reduction in chlorophyll or inhibition of chlorophyll synthesis in plants is itself a natural stress, such as plant senescence (Wang et al., 2021). Most of the reasons leading to inadequate plant productivity are attributed to the obvious weakening of plant photosynthetic capacity (An et al., 2020; Shen et al., 2021). Compared with WT plants, the Pn and Pmax of transgenic poplar were significantly reduced (Figures 6C,E,G). At the same time, the decrease in photosynthesis in transgenic plants resulted in a decrease in xylem cell layers, which slowed down the lateral growth of stems (Figures 5F–M). However, the Tr and Gs of transgenic plants were significantly higher than those of WT plants (Figures 6A,B), suggesting that downregulation of *PtrCHLP3* in plants did not inhibit photosynthesis by affecting the dark reaction process. Plant photosynthesis mainly consists of light and dark reactions, involving light absorption, electron transfer, photophosphorylation, carbon assimilation, and other important reaction steps, which are of great significance in realizing energy conversion in nature and maintaining the carbon-oxygen balance in the atmosphere (Flood et al., 2011). The photoreaction phase converts light energy into chemical energy and produces ATP, which provides energy for dark reactions. It has been reported that Fv/Fm and Y (II) reflect the maximum and actual conversion efficiencies of plant photosystem II, respectively (He et al., 2019). However, downregulation of *PtrCHLP3* in plants resulted in significantly lower Fv/Fm and Y (II) than in the WT (Figures 6H,J), which may be caused by reduced photosynthetic pigment synthesis (chlorophyll) in transgenic plants. In addition, the proportion of energy absorbed by photosystem II for photochemical reactions (qP) in transgenic plants was significantly lower than that in WT plants (Figure 6I). Concurrently, because of the low chlorophyll content of transgenic plants, they cannot carry out photosynthesis at high light intensities (Figure 6K), leading to the dissipation of excess light energy in the form of heat energy to prevent plant damage (Murchie and Niyogi, 2011). Therefore, *PtrCHLP3* mediates plant growth and development by regulating photosynthesis.

### Transcriptional Regulatory Networks Mediated by *PtrCHLP3* in Photosynthesis and Chlorophyll Biosynthesis of Poplars

As a core enzyme in chlorophyll A synthesis, *CHLP* plays a key role in plant photosynthesis and chlorophyll biosynthesis (Shpilyov et al., 2005; Lin et al., 2014). However, there are few reports on how *CHLP* functions via transcriptional regulation. Therefore, it is essential to identify the PUTFs of *PtrCHLP3*. In addition, transcriptional regulation plays a crucial role in all aspects of the plant life cycle (Zhang et al., 2022). The PUTFs of *PtrCHLP3* were identified using bioinformatic analysis (Figure 7A). Consistent with previous results (Figure 3B), the PUTFs (MYB) of *PtrCHLP3* had a tissue expression pattern similar to that of *PtrCHLP3*, and only had a high expression level in the leaves (Figure 7B). For example, the MYB TF of poplar, LTF1, promotes plant growth and xylem formation by regulating downstream target genes (Gui et al., 2019). Moreover, the trichome density and resistibility of insect pests improve due to *MYB186* overexpression in poplars, which affects plant growth (Plett et al., 2010). In short, several TFs may bind to elements such as MYB binding sites in the *CHLP* promoter, thereby regulating *CHLP* gene expression.

In plants, photosynthesis is a complex process in which a large number of genes work together to convert light energy into stable chemical energy (Flood et al., 2011). By constructing a co-expression network with the *PtrCHLP3* gene in each plant tissue (Figure 7C), a large number of genes involved in photosynthesis have been identified, including *LHCB*, *LHCA*, *PSBO*, *PSAL*, *PSBP1*, *PSAO*, *GAPA*, *ATPC*, *ATPD*, *PSAN*, and *PSBY* (Haijun Liu et al., 2007; Yi et al., 2007, 2009; Bricker and Frankel, 2008; Peng et al., 2009; Seok et al., 2014; Otani et al., 2017; Ilikova et al., 2021). For example, *LHCB3* and *LHCB6* encode light-harvesting proteins involved in photosynthesis in *Arabidopsis*, and mutations in the *LHCB3/6* in *Arabidopsis* lead to reduced plant chlorophyll content and growth inhibition (Damkjaer et al., 2009; Ilikova et al., 2021). Moreover, the three mutant (*lhcb4/5/6*) exhibited decreased energy-transfer efficiency from the LHCII (light-harvesting complex II) to the PSII reaction center (Chen et al., 2018). Similar to the phenotype of *chlp3/4* (Figures 4F, 6H), their chlorophyll content and Fv/Fm in Mutant (*lhcb4/5/6*) were also lower than the wild type. PsbO1 is a thylakoid lumen-localized extrinsic subunit protein of PSII and plays a crucial role in the oxygen-evolving complex by stabilizing the catalytic manganese cluster (Suorsa and Aro, 2007). In addition, downregulation of *PSBP* in plants showed that PS II reaction centers decreased with downregulation of PSBP composition (Yi et al., 2009). Silencing the *PtrCHLP3* gene in poplar resulted in the inhibition of chlorophyll synthesis and impaired photosynthetic ability (Figure 6). Furthermore, *LHCB1.4/3/4.2/4/6, LHCA1/2/3/4, PSBO1, PSAL, PSBP1, PSAO, GAPA, ATPC1, ATPD, PSAN*, and *PSBY* had similar expression patterns to *PtrCHLP3* and were significantly activated in leaves and involved in photosynthesis. Functional enrichment analysis showed that most of these genes, co-expressed with *PtrCHLP3*, functioned in chloroplasts and participated in plant photosynthesis (Supplementary Figure S2). In general, *PtrCHLP3* and co-expressed genes synergistically mediate photosynthesis and chlorophyll biosynthesis in poplars.

## Conclusion

**FIGURE 8:**
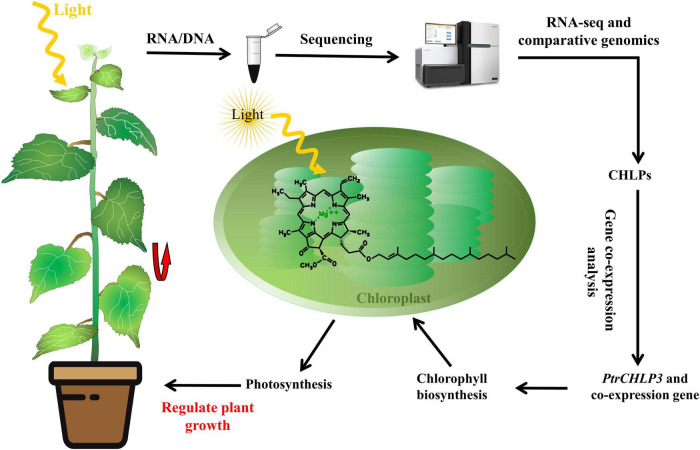
An operation model for *PtrCHLP3* regulation of poplar growth.

The data obtained in this study were used to construct a working frame to investigate whether *PtrCHLP3* regulates poplar growth (Figure 8). In summary, a whole-genome analysis of the *CHLP* family of *P. trichocarpa* identified three *PtrCHLP* genes that play pivotal roles in poplar growth and photosynthesis. The gene structure, evolution, phylogenetics, chromosomal position, gene doubling, *cis*-elements, and expression profiles of *CHLP* were determined using genomics and bioinformatics. In addition, *PtrCHLP3* is preferentially expressed in the leaves and plays an important role in regulating chlorophyll biosynthesis. Furthermore, inhibition of *PtrCHLP3* expression in poplar can inhibit plant growth through the downregulation of photosynthesis. Ultimately, *PtrCHLP3* formed a co-expression network with photosynthesis-related genes, which synergistically affected the growth and photosynthesis of the poplar trees. Thus, this study provides genetic resources for the improved breeding of fast-growing tree traits.

## Data Availability Statement

The datasets presented in this study can be found in online repositories. The names of the repository/repositories and accession number(s) can be found in the article/Supplementary Material.

## Author Contributions

FH, Y-JS, QC, J-LL, M-XN, C-HF, M-ML, Q-LL, F-FT, FZ, T-TL, L-HC, and X-QW conceived and performed the original research project. FH, Y-JS, and J-LL performed the experiments. FH, Y-JS, FZ, and X-QW designed the experiments and analyzed the data. FH refined the project and wrote the manuscript with contributions from all authors. T-TL, FZ, and X-QW supervised the experiments and revised the writing. X-QW and FH obtained the funding for the research project. All the authors read and approved the final manuscript.

## Conflict of Interest

The authors declare that the research was conducted in the absence of any commercial or financial relationships that could be construed as a potential conflict of interest.

## Publisher’s Note

All claims expressed in this article are solely those of the authors and do not necessarily represent those of their affiliated organizations, or those of the publisher, the editors and the reviewers. Any product that may be evaluated in this article, or claim that may be made by its manufacturer, is not guaranteed or endorsed by the publisher.

## Footnotes

1
https://phytozome-next.jgi.doe.gov/

2
https://www.ncbi.nlm.nih.gov/Structure/bwrpsb/bwrpsb.cgi?

3
http://gsds.gao-lab.org/

4
https://meme-suite.org/meme/tools/meme

5
http://bioinformatics.psb.ugent.be/webtools/plantcare/html/

6
https://popgenie.org/

7
http://plantregmap.gao-lab.org/network.php
