# Supplementary material for: Genome-Wide Investigation of the PtrCHLP Family Reveals That PtrCHLP3 Actively Mediates Poplar Growth and Development by Regulating Photosynthesis
Source: Front Plant Sci. 2022 May 10;13:870970. doi: 10.3389/fpls.2022.870970 (PMC9127975; doi:10.3389/fpls.2022.870970)
Supplement: Supplementary file 1 [file Data_Sheet_1.docx]

Supplementary Material

# Supplementary Tables

**Supplementary Table S1**. Primer sequences

| **Gene Name** |  | **Forward/Reverse primers** |
| --- | --- | --- |
| Primers are used to construct vectors |  |  |
| *PtrCHLP3-F1* | F | CCAGCAAGTCTAGTCCGA |
| *PtrCHLP3-R1* | R | CTTCCCATCATACTCAGT |
| Primers were used for PCR |  |  |
| *35S-F* | F | GACGCACAATCCCACTATCC |
| *RTM-R* | R | TCTATCTGCTGGGTCCAAATC |
| Primers were used for RT-PCR detection |  |  |
| *CHLP3* | F | AAACCGCAACCTCCGAGTAG |
| *CHLP3* | R | ACTCACCCACCATGCACAAA |
| *PtrActin2* | F | CCCATTGAGCACGGTATTGT |
| *PtrActin2* | R | TACGACCACTGGCATACAGG |
| *PtrUBQ* | F | AACAGCTTGAGGATGGACGA |
| *PtrUBQ* | R | TTTGCTGGTCCGGAGGGATA |

**Supplementary Table S2**. Species name and corresponding CHLP gene family name

| Order | Species | Name | Accession numbers |
| --- | --- | --- | --- |
| 1 | *A.thaliana* | *AthCHLP* | AT1G74470 |
| 2 | *G.raimondii* | *GraCHLP1* | Gorai.003G097600 |
|  |  | *GraCHLP2* | Gorai.006G119200 |
| 3 | *M.pusilla* | *MpuCHLP* | fgenesh2_kg.7_#_45_#_4270218:1 |
| 4 | *C.reinhardtii* | *CreCHLP1* | Cre01.g050950 |
|  |  | *CreCHLP2* | Cre16.g690431 |
| 5 | *T.aestivum* | *TaeCHLP* | Traes_6DL_0FF72D765 |
| 6 | *P.persica* | *PpeCHLP1* | Prupe.5G194300 |
|  |  | *PpeCHLP2* | Prupe.6G153000 |
| 7 | *V.carteri* | *VcaCHLP1* | Vocar.0001s0599 |
|  |  | *VcaCHLP2* | Vocar.0018s0244 |
| 8 | *O.sativa* | *OsaCHLP1* | LOC_Os01g16020 |
|  |  | *OsaCHLP2* | LOC_Os02g51080 |
| 9 | *A.lyrata* | *AlyCHLP* | AL2G34350 |
| 10 | *T.pratense* | *TprCHLP1* | Tp57577_TGAC_v2_gene9248 |
|  |  | *TprCHLP2* | Tp57577_TGAC_v2_gene11730 |
| 11 | *D.carota* | *DcaCHLP1* | DCAR_019413 |
|  |  | *DcaCHLP2* | DCAR_026055 |
| 12 | *P.vulgaris* | *PvuCHLP1* | Phvul.003G189600 |
|  |  | *PvuCHLP2* | Phvul.008G208000 |
| 13 | *P.deltoides* | *PdeCHLP1* | Podel.04G199300 |
|  |  | *PdeCHLP2* | Podel.09G163900 |
| 14 | *S.bicoloa* | *SbiCHLP1* | Sobic.003G122100 |
|  |  | *SbiCHLP2* | Sobic.004G238500 |
| 15 | *C.zofingiensis* | *CzoCHLP1* | Cz02g35110 |
|  |  | *CzoCHLP2* | Cz12g02030 |
| 16 | *L.sativa* | *LsaCHLP1* | Lsat_1_v5_gn_4_82121 |
|  |  | *LsaCHLP2* | Lsat_1_v5_gn_4_108620 |
|  |  | *LsaCHLP3* | Lsat_1_v5_gn_4_108640 |
|  |  | *LsaCHLP4* | Lsat_1_v5_gn_4_108780 |
|  |  | *LsaCHLP5* | Lsat_1_v5_gn_5_19701 |
| 17 | *Z.mays* | *ZmaCHLP1* | Zm00001d018034 |
|  |  | *ZmaCHLP2* | Zm00001d040356 |
| 18 | *M.sinensis* | *MsiCHLP1* | Misin07G481400 |
|  |  | *MsiCHLP2* | Misin08G270400 |
| 19 | *S.viridis* | *SviCHLP1* | Sevir.1G324600 |
|  |  | *SviCHLP2* | Sevir.5G051300 |
| 20 | *B.braunii* | *BbrCHLP* | Bobra.0352s0028 |
| 21 | *P.virgatum* | *PviCHLP1* | Pavir.1NG418900 |
|  |  | *PviCHLP2* | Pavir.1KG508900 |
|  |  | *PviCHLP3* | Pavir.5KG175900 |
|  |  | *PviCHLP4* | Pavir.5NG177000 |
| 22 | *S.purpurea* | *SpuCHLP1* | Sapur.009G123100 |
|  |  | *SpuCHLP2* | Sapur.012G049800 |
|  |  | *SpuCHLP3* | Sapur.15ZG060000 |
|  |  | *SpuCHLP4* | Sapur.15WG080500 |
| 23 | *G.hirsutum* | *GhiCHLP1* | Gohir.A03G067100 |
|  |  | *GhiCHLP2* | Gohir.D03G093600 |
|  |  | *GhiCHLP3* | Gohir.D09G101800 |
|  |  | *GhiCHLP4* | Gohir.A09G105100 |
| 24 | *P.trichocarpa* | *PtrCHLP1* | Potri.004G195800 |
|  |  | *PtrCHLP2* | Potri.009G157700 |
|  |  | *PtrCHLP3* | Potri.012G068801 |
| 25 | *D.alata* | *DalCHLP1* | Dioal.07G089200 |
|  |  | *DalCHLP2* | Dioal.19G024700 |
| 26 | *M.guttatus* | *MguCHLP* | MgTOL.B1167 |
| 27 | *T.plicata* | *TplCHLP* | Thupl.29379072s0010 |
| 28 | *S.parvula* | *SpaCHLP* | Sp5g29650 |
| 29 | *S.oleracea* | *SolCHLP* | Spov3_chr6.00959 |
| 30 | *P.acutifolius* | *PacCHLP1* | Phacu.WLD.003G218000 |
|  |  | *PacCHLP2* | Phacu.WLD.008G222700 |
| 31 | *D.strictus* | *DstCHLP* | Distr.0018s30900 |
| 32 | *C.violacea* | *CviCHLP* | Clevi.0034s0060 |
| 33 | *P.hallii* | *PhaCHLP* | Pahal.1G381100 |
| 34 | *Z.marina* | *ZmarCHLP1* | Zosma01g05020 |
|  |  | *ZmarCHLP2* | Zosma01g42560 |
| 35 | *M.esculenta* | *MesCHLP* | Manes.14G078700 |
| 36 | *P.vaginatum* | *PvaCHLP1* | Pavag03G111300 |
|  |  | *PvaCHLP2* | Pavag04G277200 |
| 37 | *F.vesca* | *FveCHLP1* | FvH4_5g14870 |
|  |  | *FveCHLP2* | FvH4_7g06120 |
| 38 | *G.max* | *GmaCHLP1* | GlymaFiskIII.02G257100 |
|  |  | *GmaCHLP2* | GlymaFiskIII.05G025500 |
|  |  | *GmaCHLP3* | GlymaFiskIII.17G098000 |
| 39 | *S.tuberosum* | *StuCHLP1* | Soltu.DM.01G026240 |
|  |  | *StuCHLP2* | Soltu.DM.03G030100 |
| 40 | *Q.rubra* | *QruCHLP1* | Qurub.01G111000 |
|  |  | *QruCHLP2* | Qurub.10G155800 |
| 41 | *S. lycopersicum* | *SlyCHLP1* | Solyc01g088310 |
|  |  | *SlyCHLP2* | Solyc03g115980.1 |
| 42 | *H.vulgare* | *HvuCHLP1* | HORVU.MOREX.r3.3HG0255220 |
|  |  | *HvuCHLP2* | HORVU.MOREX.r3.6HG0615360 |

**Supplementary Table S3**. Conserved amino acid domain

| **Gene ID** | **From** | **To** | **Short name** |
| --- | --- | --- | --- |
| *AtCHLP1* | 17 | 467 | PLN00093 superfamily |
| *OsCHLP1* | 10 | 444 | PLN00093 superfamily |
| *OsCHLP2* | 14 | 463 | PLN00093 superfamily |
| *SpCHLP2* | 16 | 470 | PLN00093 superfamily |
| *SpCHLP4* | 16 | 470 | PLN00093 superfamily |
| *SpCHLP3* | 16 | 470 | PLN00093 superfamily |
| *SpCHLP1* | 9 | 457 | PLN00093 superfamily |
| *PtrCHLP2* | 6 | 454 | PLN00093 superfamily |
| *PtrCHLP3* | 16 | 469 | PLN00093 superfamily |
| *PtrCHLP1* | 6 | 451 | PLN00093 superfamily |

| **Motif ID** | **Amino acid sequence encode** | **SeqLogo** |
| --- | --- | --- |
| Motif_1 | 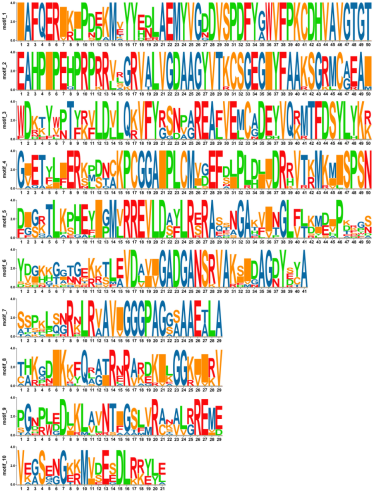IAFQERIKIPDDKMVYYENLAEMYVGDDVSPDFYGWVFPKCDHVAVGTGT |  |
| Motif_2 | EAHPIPEHPRPRRVLGRVALVGDAAGYVTKCSGEGIYFAAKSGRMCAEAI |  |
| Motif_3 | WDKTYWPTYKVLDVLQKVFYRSNPAREAFVEMCADEYVQKMTFDSYLYKK |  |
| Motif_4 | GIETYLIERKLDNCKPCGGAIPLCMVGEFDLPLDIIDRRVTKMKMISPSN |  |
| Motif_5 | DIGRTLKPHEYIGMVRREVLDAYLRERASTNGAKVINGLFLKMDIPKKGS |  |
| Motif_6 | YDGKKGGTGEKKTLEVDAVIGADGANSRVAKSIDAGDYEYA |  |
| Motif_7 | SSPKLQNRNLRVAVIGGGPAGGSAAETLA |  |
| Motif_8 | THKGDIKKFQLATRNRARDKILGGKIIRV |  |
| Motif_9 | PGNPLEDLKLAVNTIGSLVRASALRREME |  |
| Motif_10 | VEGSENGKKMVDESDLRKYLE |  |

**Supplementary Table S4**. Sequence and SeqLogo of the Motif1-1

**Supplementary Table S5**. Collinear genes among different species

| ***P. trichocarpa—A. thaliana*** | ***P. trichocarpa—S. purpurea*** | ***P. trichocarpa—P. trichocarpa*** |
| --- | --- | --- |
| *PtrCHLP3-AthCHLP1* | *PtrCHLP3-SpuCHLP2* | *PtrCHLP1-PtrCHLP2* |
|  | *PtrCHLP3-SpuCHLP3* |  |
|  | *PtrCHLP3-SpuCHLP4* |  |
|  | *PtrCHLP1-SpuCHLP1* |  |
|  | *PtrCHLP2-SpuCHLP1* |  |

**Supplementary Table S6**. Cis-acting elements on the promoters of differentially expressed target genes

| **Gene name** | **element name** | **function** | **group** |
| --- | --- | --- | --- |
| *PtrCHLP1* | W box | WRKY binding site | Abiotic and biotic stress |
| *PtrCHLP1* | W box | WRKY binding site | Abiotic and biotic stress |
| *PtrCHLP1* | RY-element | cis-acting regulatory element involved in seed-specific regulation | Growth and development |
| *PtrCHLP1* | MYC | dehydration and abscisic acid responsiveness | Abiotic and biotic stress |
| *PtrCHLP1* | MYC | dehydration and abscisic acid responsiveness | Abiotic and biotic stress |
| *PtrCHLP1* | MYC | dehydration and abscisic acid responsiveness | Abiotic and biotic stress |
| *PtrCHLP1* | MYC | dehydration and abscisic acid responsiveness | Abiotic and biotic stress |
| *PtrCHLP1* | MYC | dehydration and abscisic acid responsiveness | Abiotic and biotic stress |
| *PtrCHLP1* | GCN4_motif | cis-regulatory element involved in endosperm expression | Growth and development |
| *PtrCHLP1* | WUN-motif | wound-responsive element | Abiotic and biotic stress |
| *PtrCHLP1* | WUN-motif | wound-responsive element | Abiotic and biotic stress |
| *PtrCHLP1* | Box 4 | part of a conserved DNA module involved in light responsiveness | Growth and development |
| *PtrCHLP1* | Box 4 | part of a conserved DNA module involved in light responsiveness | Growth and development |
| *PtrCHLP1* | Box 4 | part of a conserved DNA module involved in light responsiveness | Growth and development |
| *PtrCHLP1* | TC-rich repeats | cis-acting element involved in defense and stress responsiveness | Abiotic and biotic stress |
| *PtrCHLP1* | Myb | dehydration and abscisic acid responsiveness | Abiotic and biotic stress |
| *PtrCHLP1* | LAMP-element | part of a light responsive element | Growth and development |
| *PtrCHLP1* | LAMP-element | part of a light responsive element | Growth and development |
| *PtrCHLP1* | TATC-box | cis-acting element involved in gibberellin-responsiveness | Phytohormone responsive |
| *PtrCHLP1* | ARE | cis-acting regulatory element essential for the anaerobic induction | Abiotic and biotic stress |
| *PtrCHLP1* | ARE | cis-acting regulatory element essential for the anaerobic induction | Abiotic and biotic stress |
| *PtrCHLP1* | MBSI | MYB binding site involved in flavonoid biosynthetic genes regulation | Abiotic and biotic stress |
| *PtrCHLP1* | CAG-motif | part of a light response element | Growth and development |
| *PtrCHLP1* | MYB | dehydration and abscisic acid responsiveness | Abiotic and biotic stress |
| *PtrCHLP1* | I-box | part of a light responsive element | Growth and development |
| *PtrCHLP1* | GT1-motif | light responsive element | Growth and development |
| *PtrCHLP1* | GT1-motif | light responsive element | Growth and development |
| *PtrCHLP1* | ATCT-motif | part of a conserved DNA module involved in light responsiveness | Growth and development |
| *PtrCHLP2* | TCA-element | cis-acting element involved in salicylic acid responsiveness | Phytohormone responsive |
| *PtrCHLP2* | GT1-motif | light responsive element | Growth and development |
| *PtrCHLP2* | MYB | dehydration and abscisic acid responsiveness | Abiotic and biotic stress |
| *PtrCHLP2* | MYB | dehydration and abscisic acid responsiveness | Abiotic and biotic stress |
| *PtrCHLP2* | MYB | dehydration and abscisic acid responsiveness | Abiotic and biotic stress |
| *PtrCHLP2* | MYB | dehydration and abscisic acid responsiveness | Abiotic and biotic stress |
| *PtrCHLP2* | MYB | dehydration and abscisic acid responsiveness | Abiotic and biotic stress |
| *PtrCHLP2* | MYB | dehydration and abscisic acid responsiveness | Abiotic and biotic stress |
| *PtrCHLP2* | MYB | dehydration and abscisic acid responsiveness | Abiotic and biotic stress |
| *PtrCHLP2* | STRE | stress-responsive element | Abiotic and biotic stress |
| *PtrCHLP2* | STRE | stress-responsive element | Abiotic and biotic stress |
| *PtrCHLP2* | GATA-motif | part of a light responsive element | Growth and development |
| *PtrCHLP2* | GATA-motif | part of a light responsive element | Growth and development |
| *PtrCHLP2* | ARE | cis-acting regulatory element essential for the anaerobic induction | Abiotic and biotic stress |
| *PtrCHLP2* | CAT-box | cis-acting regulatory element related to meristem expression | Growth and development |
| *PtrCHLP2* | CAT-box | cis-acting regulatory element related to meristem expression | Growth and development |
| *PtrCHLP2* | CAT-box | cis-acting regulatory element related to meristem expression | Growth and development |
| *PtrCHLP2* | Myb | dehydration and abscisic acid responsiveness | Abiotic and biotic stress |
| *PtrCHLP2* | Myb | dehydration and abscisic acid responsiveness | Abiotic and biotic stress |
| *PtrCHLP2* | Myb | dehydration and abscisic acid responsiveness | Abiotic and biotic stress |
| *PtrCHLP2* | Myb | dehydration and abscisic acid responsiveness | Abiotic and biotic stress |
| *PtrCHLP2* | Myb | dehydration and abscisic acid responsiveness | Abiotic and biotic stress |
| *PtrCHLP2* | Myb | dehydration and abscisic acid responsiveness | Abiotic and biotic stress |
| *PtrCHLP2* | TGACG-motif | cis-acting regulatory element involved in the MeJA-responsiveness | Phytohormone responsive |
| *PtrCHLP2* | AuxRR-core | cis-acting regulatory element involved in auxin responsiveness | Phytohormone responsive |
| *PtrCHLP2* | AE-box | part of a module for light response | Growth and development |
| *PtrCHLP2* | LTR | cis-acting element involved in low-temperature responsiveness | Abiotic and biotic stress |
| *PtrCHLP2* | MBS | MYB binding site involved in drought-inducibility | Abiotic and biotic stress |
| *PtrCHLP2* | MBS | MYB binding site involved in drought-inducibility | Abiotic and biotic stress |
| *PtrCHLP2* | MBS | MYB binding site involved in drought-inducibility | Abiotic and biotic stress |
| *PtrCHLP2* | MBS | MYB binding site involved in drought-inducibility | Abiotic and biotic stress |
| *PtrCHLP2* | AT1-motif | part of a light responsive module | Growth and development |
| *PtrCHLP2* | P-box | gibberellin-responsive element | Phytohormone responsive |
| *PtrCHLP2* | MRE | MYB binding site involved in light responsiveness | Growth and development |
| *PtrCHLP2* | MYC | dehydration and abscisic acid responsiveness | Abiotic and biotic stress |
| *PtrCHLP2* | MYC | dehydration and abscisic acid responsiveness | Abiotic and biotic stress |
| *PtrCHLP2* | MYC | dehydration and abscisic acid responsiveness | Abiotic and biotic stress |
| *PtrCHLP2* | MYC | dehydration and abscisic acid responsiveness | Abiotic and biotic stress |
| *PtrCHLP2* | TCT-motif | part of a light responsive element | Growth and development |
| *PtrCHLP2* | W box | WRKY binding site | Abiotic and biotic stress |
| *PtrCHLP2* | W box | WRKY binding site | Abiotic and biotic stress |
| *PtrCHLP2* | RY-element | cis-acting regulatory element involved in seed-specific regulation | Growth and development |
| *PtrCHLP2* | CGTCA-motif | cis-acting regulatory element involved in the MeJA-responsiveness | Phytohormone responsive |
| *PtrCHLP3* | GC-motif | enhancer-like element involved in anoxic specific inducibility | Abiotic and biotic stress |
| *PtrCHLP3* | AE-box | part of a module for light response | Growth and development |
| *PtrCHLP3* | LTR | cis-acting element involved in low-temperature responsiveness | Abiotic and biotic stress |
| *PtrCHLP3* | ABRE | cis-acting element involved in the abscisic acid responsiveness | Phytohormone responsive |
| *PtrCHLP3* | Sp1 | light responsive element | Growth and development |
| *PtrCHLP3* | MBS | MYB binding site involved in drought-inducibility | Abiotic and biotic stress |
| *PtrCHLP3* | MBS | MYB binding site involved in drought-inducibility | Abiotic and biotic stress |
| *PtrCHLP3* | MYC | dehydration and abscisic acid responsiveness | Abiotic and biotic stress |
| *PtrCHLP3* | MYC | dehydration and abscisic acid responsiveness | Abiotic and biotic stress |
| *PtrCHLP3* | MYC | dehydration and abscisic acid responsiveness | Abiotic and biotic stress |
| *PtrCHLP3* | MYC | dehydration and abscisic acid responsiveness | Abiotic and biotic stress |
| *PtrCHLP3* | MYC | dehydration and abscisic acid responsiveness | Abiotic and biotic stress |
| *PtrCHLP3* | G-box | cis-acting regulatory element involved in light responsiveness | Growth and development |
| *PtrCHLP3* | chs-CMA1a | part of a light responsive element | Growth and development |
| *PtrCHLP3* | W box | WRKY binding site | Abiotic and biotic stress |
| *PtrCHLP3* | TCT-motif | part of a light responsive element | Growth and development |
| *PtrCHLP3* | TCT-motif | part of a light responsive element | Growth and development |
| *PtrCHLP3* | MYB | dehydration and abscisic acid responsiveness | Abiotic and biotic stress |
| *PtrCHLP3* | MYB | dehydration and abscisic acid responsiveness | Abiotic and biotic stress |
| *PtrCHLP3* | MYB | dehydration and abscisic acid responsiveness | Abiotic and biotic stress |
| *PtrCHLP3* | MYB | dehydration and abscisic acid responsiveness | Abiotic and biotic stress |
| *PtrCHLP3* | MYB | dehydration and abscisic acid responsiveness | Abiotic and biotic stress |
| *PtrCHLP3* | GT1-motif | light responsive element | Growth and development |
| *PtrCHLP3* | GT1-motif | light responsive element | Growth and development |
| *PtrCHLP3* | GT1-motif | light responsive element | Growth and development |
| *PtrCHLP3* | GT1-motif | light responsive element | Growth and development |
| *PtrCHLP3* | GT1-motif | light responsive element | Growth and development |
| *PtrCHLP3* | Gap-box | part of a light responsive element | Growth and development |
| *PtrCHLP3* | I-box | part of a light responsive element | Growth and development |
| *PtrCHLP3* | I-box | part of a light responsive element | Growth and development |
| *PtrCHLP3* | Myc | dehydration and abscisic acid responsiveness | Abiotic and biotic stress |
| *PtrCHLP3* | ARE | cis-acting regulatory element essential for the anaerobic induction | Abiotic and biotic stress |
| *PtrCHLP3* | ARE | cis-acting regulatory element essential for the anaerobic induction | Abiotic and biotic stress |
| *PtrCHLP3* | ARE | cis-acting regulatory element essential for the anaerobic induction | Abiotic and biotic stress |
| *PtrCHLP3* | ARE | cis-acting regulatory element essential for the anaerobic induction | Abiotic and biotic stress |
| *PtrCHLP3* | STRE | stress-responsive element | Abiotic and biotic stress |
| *PtrCHLP3* | STRE | stress-responsive element | Abiotic and biotic stress |
| *PtrCHLP3* | STRE | stress-responsive element | Abiotic and biotic stress |
| *PtrCHLP3* | STRE | stress-responsive element | Abiotic and biotic stress |
| *PtrCHLP3* | GATA-motif | part of a light responsive element | Growth and development |
| *PtrCHLP3* | GATA-motif | part of a light responsive element | Growth and development |
| *PtrCHLP3* | G-Box | cis-acting regulatory element involved in light responsiveness | Growth and development |
| *PtrCHLP3* | Myb | dehydration and abscisic acid responsiveness | Abiotic and biotic stress |
| *PtrCHLP3* | Myb | dehydration and abscisic acid responsiveness | Abiotic and biotic stress |
| *PtrCHLP3* | GA-motif | part of a light responsive element | Growth and development |
| *PtrCHLP3* | TC-rich repeats | cis-acting element involved in defense and stress responsiveness | Abiotic and biotic stress |
| *PtrCHLP3* | LAMP-element | part of a light responsive element | Growth and development |
| *PtrCHLP3* | TCCC-motif | part of a light responsive element | Growth and development |
| *PtrCHLP3* | TCCC-motif | part of a light responsive element | Growth and development |
| *PtrCHLP3* | Box 4 | part of a conserved DNA module involved in light responsiveness | Growth and development |
| *PtrCHLP3* | Box 4 | part of a conserved DNA module involved in light responsiveness | Growth and development |

**Supplementary Table S7**. Expression of *PtrCHLP* genes in diverse poplar tissues

| gene_id | Buds Dormant | Buds Pre chilling | Flowers Dormant | Flowers Expanded | Flowers Expanding | Leaves Freshly Expanded | Leaves Mature | Leaves Young Expanding | Roots | Seeds Mature | Suckers | Wood |
| --- | --- | --- | --- | --- | --- | --- | --- | --- | --- | --- | --- | --- |
| PtrCHLP1 | 0.72 | 0.81 | 1.37 | -0.85 | 0.84 | -1.35 | -0.43 | 0.28 | 2.03 | -0.81 | 0.38 | 2.12 |
| PtrCHLP2 | 0.51 | 0.73 | 1.28 | 0.75 | 0.29 | -0.03 | -1.17 | 0.12 | 1.1 | 1.25 | 0.1 | -1.58 |
| PtrCHLP3 | -0.64 | -0.59 | 0.13 | -0.91 | -0.13 | 0.8 | 1.23 | 0.32 | -4.79 | -1.13 | 1.38 | -3.79 |

**Supplementary Table S8**. Upstream transcription factors of *PtrCHLP3*

| **TF** | **Method** | **Position** | **Strand** | **Sequence** | **DistanceToTSS** | **P-value** |
| --- | --- | --- | --- | --- | --- | --- |
| Potri.012G060300(MYBS1) | motif | Chr12:9143374-9143388 | + | AAAACCTTATCCACC | -49 | 7.06E-06 |
| Potri.012G073900(MYB_related) | motif | Chr12:9143498-9143512 | + | AAACCCAACCGTAAC | 61 | 4.20E-06 |
| Potri.010G240800(DIV2) | motif | Chr12:9143378-9143387 | - | GTGGATAAGG | -50 | 1.00E-06 |
| Potri.008G191800(MYBD) | motif | Chr12:9143377-9143386 | - | TGGATAAGGT | -51 | 5.31E-06 |
| Potri.002G228700(MYB101) | motif | Chr12:9143373-9143387 | + | CAAAACCTTATCCAC | -50 | 4.06E-08 |
| Potri.001G155300(MYBH) | motif | Chr12:9143376-9143389 | - | TGGTGGATAAGGTT | -48 | 1.63E-06 |

**Supplementary Table S9**. Transcription levels of upstream transcription factors of *PtrCHLP3*

|  | Potri.012G060300 | Potri.012G073900 | Potri.010G240800 | Potri.008G191800 | Potri.002G228700 | Potri.001G155300 |
| --- | --- | --- | --- | --- | --- | --- |
| Mature Leaves | 2.01 | 0.49 | -0.13 | 0.53 | 0.31 | -0.08 |
| Young Leaves | 0.28 | 0.41 | -0.23 | -0.52 | 0 | -0.11 |
| Stem | 0 | -0.07 | 0 | -0.36 | 0.1 | 0.17 |
| Roots | -0.05 | 0 | 1.43 | 0.73 | -0.26 | 0.41 |
| Stem Nodes | -0.34 | -0.18 | 0.1 | 0 | -0.24 | 0 |

# Supplementary Figures

#
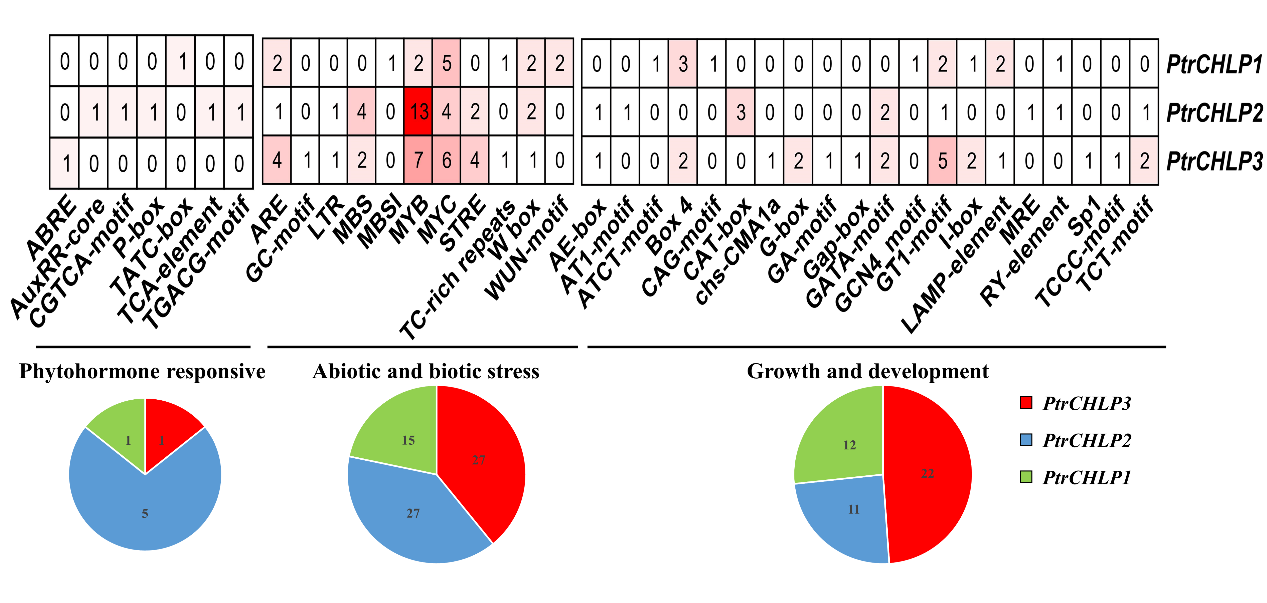
**Supplementary Figure S1.** Analysis of cis-acting regulatory elements of target gene promoter of PtrCHLPs. The gradient colors in the red grid represent the number of cis-acting elements in PtrCHLPs. The multicolored pie chart indicates the cis-elements in each category. These elements are divided into three categories in terms of the functional notes: plant hormone response, abiotic stress and biotic stress, and plant growth and development.


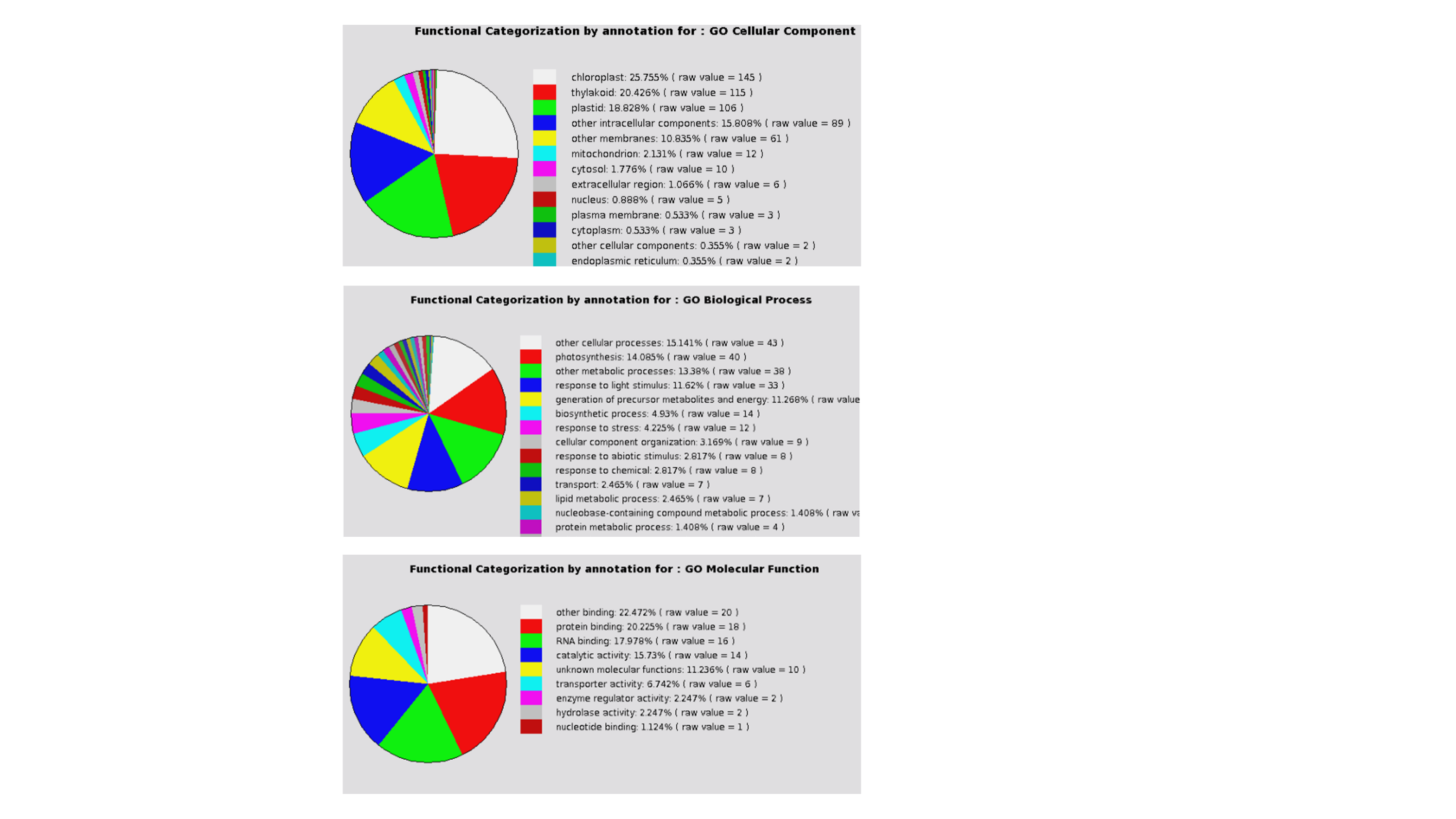


**Supplementary Figure S2.** GO enrichment analysis of co-expressing genes with *PtrCHLP3*, including GO cellular components, GO biological processes, and GO molecular functions.

**Supplementary Figure S3.** The absolute expression of *PtrCHLP1*/2/3 in different plant tissues at various growth stages.
